# North Dakota State Veterinary Medical Association

**Published:** 1892-05

**Authors:** W. T. Crew

**Affiliations:** Secretary


*North Dakota State Veterinary Medical Association.*—The veterinarians of North Dakota met at Hillsboro, April 1st, and organized a State Veterinary Medical Association. The following officers were chosen for the ensuing year: President, T. D. Hinebauch, M. S. V. S., N. D. Agricultural College, Fargo; Vice-President, E. H. Rishel, D. V. S., Mayville; Secretary, W. T. Crew, V. S., Reynolds; Treasurer, B. C. Taylor, V. S., Hillsboro.

Committees were appointed to draft Constitution and By-laws, to be reported on at the next meeting, which will be held in Fargo, during June, date to be announced later.

*Dr. Hinebauch presented a paper describing an outbreak which occurred during the fore-part of the winter. The paper was thoroughly discussed. *Dr. Farmer:* How long after the carrots were eaten before a post mortem was had? *Dr. Crew:* Five days. Two days after they were eaten the first animals became attacked. They did not all show the same symptoms. One symptom which, however, was invariably present, and the first to attract attention, was the loss of the power of deglutition. *Dr. Farmer:* That would indicate lesions of the medulla

*Paper is printed in full.

oblongata. *Dr. Taylor :* In this case we found no bots in the stomach, but there were a great many in the first six inches of the duodenum, probably driven out by bile. The mucous membrane of the cardiac portion of the stomach was partially denuded. *Dr. Tully :* The character of the secretions was changed and undoubtedly caused this denuding. *Dr. Farmer :* Do you think, from the appearance of the exudation around the cord, that it was of rapid origin? *Dr. Hinebauch :* It would be hard to give a correct answer to such question. However, if secretions are rapidly formed they are usually of a lighter color than when slowly formed. When slowly formed they are usually of a deep amber color, and in this case there had been some exudation of blood, so that the secretions were discolored and no definite answer could be given. I think, however, that the history of the case, and the length of time in which the disease run its course would indicate that the secretion must have taken place very rapidly or the symptoms could not have followed one another in so short a time. *Dr. Farmer :* Are you sure that the worms you describe are the *Oxyuris Vermicularis*? Our attention, during the past winter was called to a similar condition down in Illinois; in that case the worms were the *Tetracanthis*. *Dr. Hinebauch :* We have some of the specimens right here, and I will place them under the microscope and allow you to examine them, so that you can satisfy yourselves with regard to the variety. After having made a thorough examination, what conclusion have you arrived at? *Dr. Farmer:* These, certainly, are the variety you describe, but I certainly do not understand how they could have gotten into the muscles and abdominal cavity. *Dr. Hinebauch :* They were there, nevertheless, in countless numbers. I have been sorry since then that I did not examine thoroughly the larger blood vessels, to see whether they were not also present there. It did not enter my mind at the time that they might exist in such places. I have also been advised of the outbreak that occurred in Illinois, it happening on the farm of a Mr. Baker, of Manhattan, who sent me a number of the worms. He also stated that they existed in the blood vessels, that they were very numerous there, and when I come to compare them with these. I find they are identical, both belonging to the same variety. I think, however, that is an unusual position for these worms. W. T. CREW, *Secretary.*

